# Connecting Hazard Analysts and Risk Managers to Sensor Information

**DOI:** 10.3390/s8063932

**Published:** 2008-06-11

**Authors:** Gonéri Le Cozannet, Steven Hosford, John Douglas, Jean-Jacques Serrano, Damien Coraboeuf, Jérémie Comte

**Affiliations:** 1 BRGM - French Geological Survey, 3 avenue Claude Guillemin, 45060 Orléans, France; E-mails: j.douglas@brgm.fr (J.D.); jj.serrano@brgm.fr (J.-J.S.); dcoraboeuf@yahoo.fr (D.C.); j.comte@brgm.fr (J.C.); 2 CNES, 18, avenue Edouard Belin, 31401 Toulouse cedex 9, France; E-Mail: steven.hosford@cnes.fr (S.H.)

**Keywords:** hazard maps, geohazards, OGC metadata catalogue, risk management, GEOSS

## Abstract

Hazard analysts and risk managers of natural perils, such as earthquakes, landslides and floods, need to access information from sensor networks surveying their regions of interest. However, currently information about these networks is difficult to obtain and is available in varying formats, thereby restricting accesses and consequently possibly leading to decision-making based on limited information. As a response to this issue, state-of-the-art interoperable catalogues are being currently developed within the framework of the Group on Earth Observations (GEO) workplan. This article provides an overview of the prototype catalogue that was developed to improve access to information about the sensor networks surveying geological hazards (geohazards), such as earthquakes, landslides and volcanoes.

## 1. Introduction

Attempts to catalogue sensor data lead to gathering heterogeneous information, which makes the architecture of such catalogues difficult to manage. This is because scientists and engineers concerned with geological hazards, such as earthquakes, landslides and volcanoes (here grouped under the collective term *geohazards*), use heterogeneous in-situ and remote sensing data and modelling tools to produce information for decision makers. Hazard maps are one of the final products that land-use planners need during the planning and development of new buildings and infrastructure. In order to produce hazard maps, scientists need various sensor data from varied sources including geological maps, databases of historical events, digital elevation models, as well as land-cover maps. This information can be derived from data produced by many types of sensors, such as high-resolution space or airborne imagery, hyper-spectral data, borehole data, seismometer data, GPS networks and other geophysical in-situ and space instrumentation. While it is very challenging to produce a metadata system gathering all data available for hazard mapping, another approach, which consists of collecting the existing hazard maps and their underlying data, is proposed here.

## 2. International framework

The design of a Global Earth Observing System of Systems (GEOSS) to respond to society's needs for information on the earth environment has been the concern of GEO, the Group on Earth Observations (http://www.earthobservations.org/), since its inception as an *ad hoc* group in July 2003. One of the key challenges of GEOSS is its architecture. The users of GEOSS will expect to use a flexible system, enabling search, retrieval and archiving of accessible datasets through a distributed network of servers. In order to achieve this goal, interoperability arrangements amongst data providers are necessary. This implies widespread adoption of state-of-the-art interoperability standards.

GEO's “Geohazards Community of Practice” (CoP, http://www.igosgeohazards.org/), is an international partnership of space agencies, geological surveys and scientific organisations that are concerned with improving access to geospatial data and information for the mitigation, prevention and monitoring of geophysical hazards. This community undertook, under GEO, to design and develop an interoperable and distributed metadata system applied to the inventory of hazard maps. This system has been named GeoHazData.

## 3. GeoHazData concept

During the 2006-2007 period, the GEO Geohazards CoP was used to collect user requirements. Then, a metadata editor and a web map service were produced, and finally, strategies to perform the actual inventory were tested.

### User requirements

GeoHazData takes as its starting point an end user need: the requirement to access geohazard maps for a region. The requirement of decision makers for hazard evaluation is common to all geohazards. Hazard is commonly characterised in terms of the probability of a measurable physical parameter (or, occasionally, parameters) exceeding a certain threshold during a period of time. For example, the conference proceedings edited by Vecchia [1] shows that this is a common approach for many natural hazards. In addition, Douglas [2] shows that there are many common requirements and procedures for the development and presentation of such hazard (and, when hazard is combined with a consideration of vulnerability, risk) maps. However, even though the overall goal of most geohazard assessments is similar, the methods for their production and the data used are generally peril-specific.

**Figure 1.**
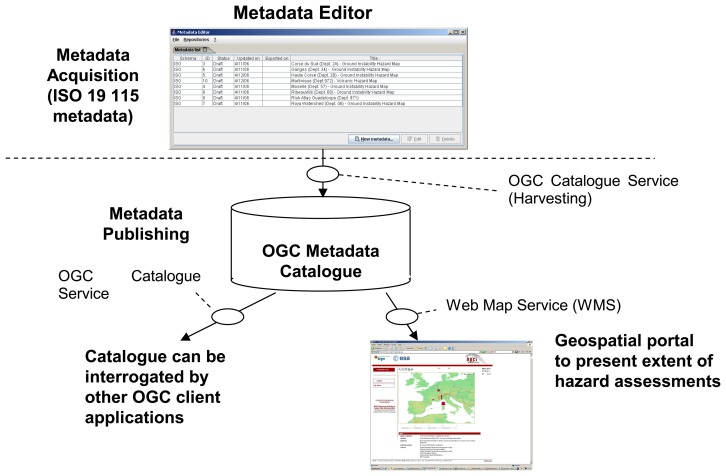
Overall concept of the GeoHazData hazard map inventory tool. The metadata editor generates ISO 19115-compatible metadata registered in an Open Geospatial Consortium (OGC, http://www.opengeospatial.org/) catalogue [5]. This catalogue is stored on a publicly accessible server. The acquired data can be shared using a dedicated server, published on any other OGC compliant catalogue or exported locally in XML format. A Web Map Service (WMS) enables viewing the extent and content of the metadata. Finally, this catalogue can be interrogated by any other OGC client application.

The analysis of the needs of potential users of GeoHazData was performed in order to produce a metadata system able to describe the hazards maps in a homogeneous way [4]. During the 2^nd^ International Geohazards Workshop held at BRGM in Orléans (France) in June 2005, a working group of scientists and experts concerned with integration of data to produce seamless information for decision makers was set up, with the aim of identifying the data used to produce information products such as hazard maps. This working group produced a preliminary user requirement document, which was reviewed and completed by a survey launched in early 2006. This user requirement process allowed the identification of the required fields and definitions of the hazard map inventory system. Figure 1 gives an overview of the overall concept of the GeoHazData hazard map inventory tool. It includes the following components.

### GeoHazData Architecture

### Catalogue content

The user requirements process showed that the critical parameters for the characterization of hazard maps are the following.

- Fundamental data: pertaining to the characteristics of the map itself (e.g. coverage, scale, date of production, creator and publishing organisation). These are “standard” data fields that are relevant to many geospatial datasets and are already provided for in geospatial metadata standards.
- Information providing an indication of the quality of the map including how and why this map was generated and the data and methods used.

One of the important user requirements was to enable GeoHazData users to access information on the source data, i.e. to the data layers as well as to the processes used to produce this hazard map. This helps the user understand the “reliability” of the hazard evaluation and gives an indication of how the hazard map should be used. This is characterized by:

- The sources: the user requirements process defined a set of “interpreted data layers” for each of the geohazards that are typically required for evaluating hazard. These interpreted data layers are listed in Table 1. For each of these interpreted data layers a certain number of measured data layers have been identified.
- The process: that allowed the production of the hazard maps. The process is often a clearly defined process, such as modelling tools used to map the hazard complemented by advice from geologists.
- Legal constraints on access and use applied to the hazard map itself or of the underlying data.

### Interoperability features

In order to be a useful contribution to GEOSS, GeoHazData must be interoperable with other catalogues contributing to GEOSS. As an OGC catalogue of ISO 19115 metadata GeoHazData uses state-of-the-art interoperability standards and is, therefore, interoperable with other OGC catalogues and clients. This follows the recommendations of the GEO Data and Architecture Committee. The interoperability features of GeoHazData were successfully tested during a demonstration of the GEOSS Clearing House at GEO III (Bonn) in November 2005. This demonstration showed however that clients require some specific adjustments to be able to access in an interoperable way catalogues based on various profiles.

## Lessons learnt and conclusion

The GeoHazData hazard map inventory tool was intended as a contribution to the implementation of GEOSS. In order to test this concept, some metadata were added to the server using the metadata editor. These metadata mostly come from previous hazard surveys performed by BRGM.

The most critical issue for the actual implementation of the GEOSS Geohazards component is to find a cooperation scheme under which the members actually commit to insert content in the database. GEO and its Geohazards CoP were identified as the appropriate frameworks. Our approach was to propose the GeoHazData concept to GEO Member states in order to launch the implementation process. GEO member states and participating organisations are invited to critically review the GeoHazData hazard map inventory tool.

## Figures and Tables

**Table 1.** List of sources useful for the creation of hazard maps. These sources allow the user to identify the data available for the region of interest. Volcanic hazards correspond to all hazards known in the area of interest. These can be (non-exhaustive list): lava flows, ash fall, gas emissions, lahars, ground movements, tidal waves or hydrothermal explosion. Volcanic hazard maps either show the estimated probability of occurrence of one of these hazards only, or a combination of all these hazards. As an example, a volcanic multi-risk approach has been described in Thierry et al., 2007 [3].

**Seismic hazard**	**Ground instability hazard**	**Volcanic hazards**

Instrumental seismicity catalogue	Geological characterisation	Volcanological structure
Amplification spectrum	Geotechnical characterisation	Geological characterisation
Crustal deformation in region	Hydrogeological characterisation	Geomorphological characterisation
Active fault catalogue	Geomorphological characterisation	
Model of crustal velocity structure	Topographic surface	Geotechnical characterisation
*A posteriori* liquefaction occurrence during earthquake	Deformation monitoring	Hydrogeological characterisation
		Gas information
Liquefaction susceptibility		Historical events database
Earthquake forecast maps		Deformation monitoring
Digital elevation model (site effects)		Geochemical monitoring
		Seismic monitoring
